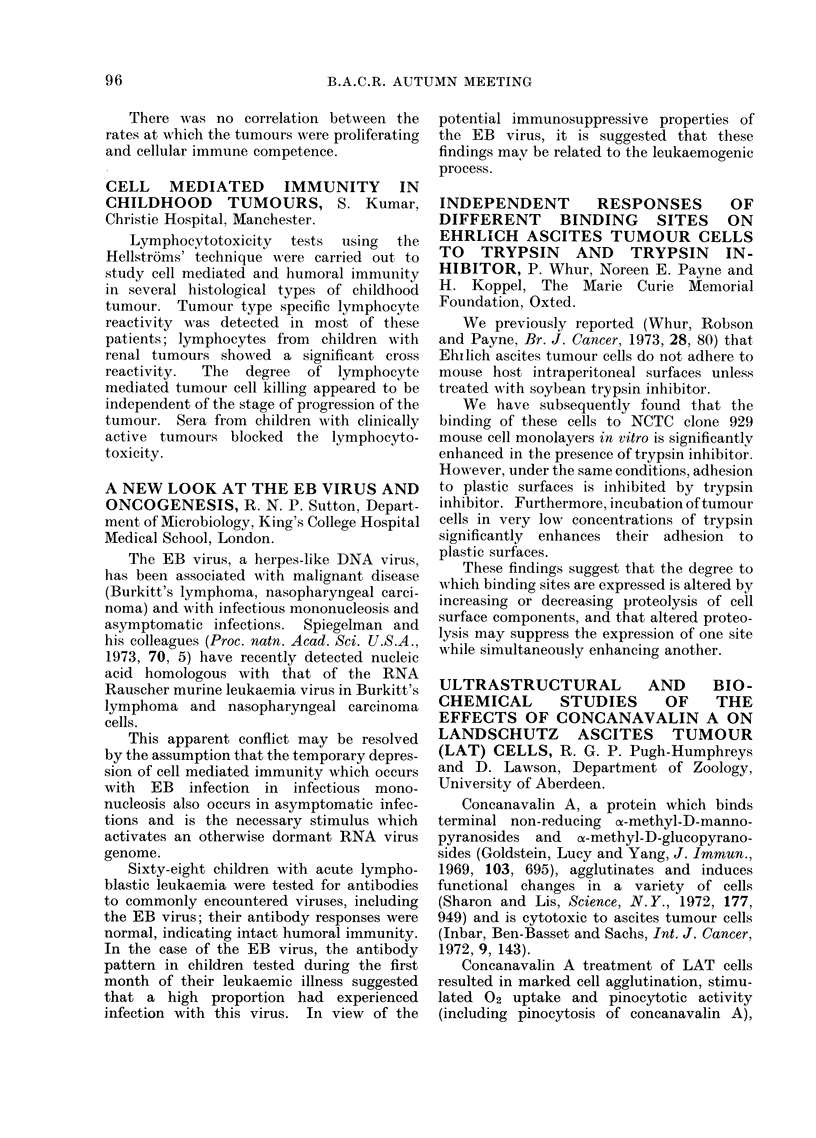# Proceedings: A new look at the EB virus and oncogenesis.

**DOI:** 10.1038/bjc.1974.27

**Published:** 1974-01

**Authors:** R. N. Sutton


**A NEW LOOK AT THE EB VIRUS AND ONCOGENESIS,** R. N. P. Sutton, Department of Microbiology, King's College Hospital Medical School, London.

The EB virus, a herpes-like DNA virus, has been associated with malignant disease (Burkitt's lymphoma, nasopharyngeal carcinoma) and with infectious mononucleosis and asymptomatic infections. Spiegelman and his colleagues (*Proc. natn. Acad. Sci. U.S.A.*, 1973, **70**, 5) have recently detected nucleic acid homologous with that of the RNA Rauscher murine leukaemia virus in Burkitt's lymphoma and nasopharyngeal carcinoma cells.

This apparent conflict may be resolved by the assumption that the temporary depression of cell mediated immunity which occurs with EB infection in infectious mononucleosis also occurs in asymptomatic infections and is the necessary stimulus which activates an otherwise dormant RNA virus genome.

Sixty-eight children with acute lymphoblastic leukaemia were tested for antibodies to commonly encountered viruses, including the EB virus; their antibody responses were normal, indicating intact humoral immunity. In the case of the EB virus, the antibody pattern in children tested during the first month of their leukaemic illness suggested that a high proportion had experienced infection with this virus. In view of the potential immunosuppressive properties of the EB virus, it is suggested that these findings may be related to the leukaemogenic process.